# Rosiglitazone Suppresses Renal Crystal Deposition by Ameliorating Tubular Injury Resulted from Oxidative Stress and Inflammatory Response via Promoting the Nrf2/HO-1 Pathway and Shifting Macrophage Polarization

**DOI:** 10.1155/2021/5527137

**Published:** 2021-10-14

**Authors:** Hongyan Lu, Xifeng Sun, Min Jia, Fa Sun, Jianguo Zhu, Xiaolong Chen, Kun Chen, Kehua Jiang

**Affiliations:** ^1^Department of Urology, The Third Affiliated Hospital of Chongqing Medical University, Chongqing 401120, China; ^2^Department of Urology, Medical University of Graz, Graz 8036, Austria; ^3^Department of Urology, Guizhou Provincial People's Hospital, Guiyang 550002, China; ^4^Prenatal Diagnosis Center, Guizhou Provincial People's Hospital, Guiyang 550002, China; ^5^Medical College of Guizhou University, Guiyang, China

## Abstract

Oxidative stress and inflammatory response are closely related to nephrolithiasis. This study is aimed at exploring whether rosiglitazone (ROSI), a regulator of macrophage (Mp) polarization, could reduce renal calcium oxalate (CaOx) deposition by ameliorating oxidative stress and inflammatory response. Male C57 mice were equally and randomly divided into 7 groups. Kidney sections were collected on day 5 or day 8 after treatment. Pizzolato staining and polarized light optical microscopy were used to detect crystal deposition. PAS staining and TUNEL assay were performed to assess the tubular injury and cell apoptosis, respectively. Gene expression was assessed by immunohistochemistry, immunofluorescence, ELISA, qRT-PCR, and Western blot. The reactive oxygen species (ROS) level was assessed using a fluorescence microplate and fluorescence microscope. Hydrogen peroxide (H_2_O_2_), malonaldehyde (MDA), and glutathione (GSH) were evaluated to determine oxidative stress. Lactic dehydrogenase (LDH) activity was examined to detect cell injury. Adhesion of CaOx monohydrate (COM) crystals to HK-2 cells was detected by crystal adhesion assay. HK-2 cell death or renal macrophage polarization was assessed by flow cytometry. *In vivo*, renal crystal deposition, tubular injury, crystal adhesion, cell apoptosis, oxidative stress, and inflammatory response were significantly increased in the 7-day glyoxylic acid- (Gly-) treated group but were decreased in the ROSI-treated groups, especially in the groups pretreated with ROSI. Moreover, ROSI significantly reduced renal Mp aggregation and M1Mp polarization but significantly enhanced renal M2Mp polarization. In vitro, ROSI significantly suppressed renal injury, apoptosis, and crystal adhesion of HK-2 cells and markedly shifted COM-stimulated M1Mps to M2Mps, presenting an anti-inflammatory effect. Furthermore, ROSI significantly suppressed oxidative stress by promoting the Nrf2/HO-1 pathway in HK-2 cells. These findings indicate that ROSI could ameliorate renal tubular injury that resulted from oxidative stress and inflammatory response by suppressing M1Mp polarization and promoting M2Mp polarization. Therefore, ROSI is a potential therapeutic and preventive drug for CaOx nephrolithiasis.

## 1. Introduction

Urolithiasis has a reported prevalence of 10% and 50% recurrence rates [1], potentially resulting in chronic kidney disease or even renal failure [2]. In addition, it has been estimated that, by 2030, the annual cost for managing urolithiasis in the United States is estimated to be about 4.6 billion dollars [3].

In the process of calcium oxalate (CaOx) crystal deposition, injury to the tubular epitheliums [4], which is frequently mediated by oxidative stress, plays a critical role during CaOx crystal deposition [5]. CaOx crystals upregulate NADPH oxidase p47phox and lead to overproduction of reactive oxygen species (ROS) in the tubular epitheliums. This is further enhanced by the proinflammatory effect of macrophages [6]. ROS are the leading mediators of oxidative stress injury, damaging the mitochondrial membrane and reducing the transmembrane potential [7, 8]. In addition, damaged tubular cells can act as the adhesive site for crystals, increasing their adhesion [9]. Therefore, CaOx crystal-induced oxidative stress is a critical process for nephrolithiasis formation.

Inflammation is another crucial process in nephrolithiasis formation. Following exposure to crystals, renal epitheliums express increased levels of monocyte chemotactic protein-1 (MCP1), inducing substantial Mp recruitment [10]. Mps play pivotal roles in inflammation and display different functional roles when responding to diverse microenvironmental signals [11, 12]. Two main phenotypes of Mps have been found: classically activated M1Mps, which are proinflammatory, and alternatively activated M2Mps, known to demonstrate an anti-inflammatory effect. CaOx crystals promoted M1Mp polarization which worsens the renal condition, resulting in fibrosis and chronic kidney disease [13, 14]. M1Mps reportedly promote renal crystal deposition in the mouse model [5]. On the contrary, stimulating M2Mp polarization could reduce renal injury [15–17]. Moreover, Mps have plasticity, by which polarized M1Mps can shift to M2Mps by employing specific signals [18]. Consequently, the M1/M2 Mp phenotype shift plays a pivotal role in regulating inflammation.

Nuclear factor erythroid-2-related factor 2 (Nrf2) is an antioxidative mediator which acts by regulating downstream genes [19, 20], including heme oxygenase 1 (HO-1). The Nrf2/HO-1 pathway is one of the most classical antioxidative approaches [21].

Peroxisome proliferator-activated receptor *γ* (PPAR*γ*) is a nuclear receptor and inflammatory regulator [22]. PPAR*γ* activation plays an anti-inflammatory effect by repressing intranuclear signaling pathways, containing nuclear factor-*κ*B (NF-*κ*B) in Mps [23], and has an antioxidant effect [24]. Moreover, PPAR*γ* activation is critical for the polarization and maintenance of M2Mps [25, 26]. These characteristics indicate that PPAR*γ* agonists might be a promising therapeutic option for nephrolithiasis.

However, data regarding the antioxidant and anti-inflammatory activities of PPAR*γ* agonist ROSI are greatly limited. This study revealed that ROSI could suppress crystal deposition in the kidney of hyperoxaluric mice by inhibiting oxidative stress and inflammatory response.

## 2. Materials and Methods

### 2.1. Reagents

Glyoxylic acid (Gly) was acquired from Sigma-Aldrich (St. Louis, MO). COM crystals were prepared by the chemical method as described previously [27] and used in in vitro experiments at a concentration of 300 *μ*g/mL. PPAR*γ* agonist ROSI and antagonist GW9662 were both procured from MedChemExpress (MCE, China).

### 2.2. Animal Experiments

All animal experiments fulfilled the criteria of NIH and Guizhou Provincial People's Hospital for the humane treatment of laboratory animals and were approved by the Animal Care and Use Committee of Guizhou Provincial People's Hospital. Male C57BL/6 mice aged 6–8 weeks were acquired from the Experimental Animal Center and reared in SPF animal facilities at Guizhou Provincial People's Hospital.

In order to assess the effects of ROSI, 42 mice were equally assigned to 7 groups: the control group, 4 days 80 mg/kg Gly (Gly 4d) group, 7 days 80 mg/kg Gly (Gly 7d) group, 7 days 80 mg/kg Gly plus 2.5 mg/kg or 5 mg/kg ROSI without pretreatment of ROSI (Gly+ROSI 7d(L)) group, Gly+ROSI 7d(H)) group, and 7 days 80 mg/kg Gly plus 2.5 mg/kg or 5 mg/kg ROSI with pretreatment of 3 days 2.5 mg/kg or 5 mg/kg ROSI (ROSI 3d+Gly+ROSI 7d(L), and ROSI 3d+Gly+ROSI 7d(H)) group. Mice were given a daily intraabdominal injection with Gly or vehicle (phosphate-buffered saline (PBS), Gibco) for 4 or 7 days. ROSI or vehicle (PBS) was administered via the gastric tube once daily. The kidneys were acquired on day 5 or day 8 after treatment to detect crystal deposition and for other related experiments.

### 2.3. Cell Culture and Treatment

THP-1 and HK-2 cells were purchased from the Cell Bank of the Chinese Academy of Science (Shanghai, China). THP-1 cells were cultured in RPMI-1640 (Gibco, USA) conditioned medium, and HK-2 cells were cultured in DMEM/F12 (Gibco, USA) conditioned medium containing 10% FBS and 1% penicillin-streptomycin in an incubator at 37°C and 5% CO_2_.

For differentiating into M0Mps, THP-1 cells were diluted into a density of 2 × 10^5^/mL and treated with 10 ng/mL phorbol-12-myristate-13-acetate (Sigma-Aldrich, USA) for 24 h. After removing the supernatant, the cells were washed thrice with PBS and cultured in RPMI-1640 conditioned medium for subsequent experiments.

For activating or suppressing PPAR*γ*, THP-1 and HK-2 cells were treated with 1 *μ*M ROSI or 10 *μ*M GW9662, respectively, for 48 h. COM crystals (300 *μ*g/mL) were utilized to treat cells for 48 h. HK-2 cells were transfected with Nrf2-siRNA for silencing Nrf2 expression.

### 2.4. Observation of the Deposition of Renal CaOx Crystal

Crystal deposition in sections was examined through Pizzolato staining, as presented previously [28]. Crystal containing calcium was determined through polarized light optical microscopy (CX31P; Olympus, Japan) in unstained sections. Crystal deposition was assessed quantitatively by ImageJ (National Institute of Health, USA) to calculate the percentage of the crystal deposition area in the entire kidney section or corticomedullary border.

### 2.5. Immunohistochemistry (IHC) and Immunofluorescence (IF)

Briefly, kidney samples were fixed for 24 h with 4% buffered formalin before embedding into paraffin. Subsequently, sections at 4 *μ*m thick were acquired. For IHC, the slides were incubated within antibodies for PPAR*γ* (1 : 150, 2435, CST), iNOS (1 : 400, AF0199, Affinity), Arg1 (1 : 150, DF6657, Affinity), F4/80 (1 : 150, DF7762, Affinity), MCP1 (1 : 150, DF7577, Affinity), OPN (1 : 100, AF0227, Affinity), CD44 (1 : 100, DF6392, Affinity), IL-1*β* (1 : 150, AF5103, Affinity), HO-1 (1 : 200, AF5393, Affinity), Nrf2 (1 : 100, AF0639, Affinity), and SOD1 (1 : 150, AF5198, Affinity). Kidney sections were quantified through evaluating the proportion of the positive areas to the entire kidney section by ImageJ.

Immunoreactivity was examined by Histofine Simple Stain Kit for rabbit IgG following the protocol from the manufacturer. The Mp phenotypes were determined by IF staining for iNOS (1 : 100, 13120, CST) and Arg1 (1 : 100, 93668, CST). The slides of THP-1 cells were fixed for 15 minutes in 4% buffered formalin. After being rinsed with PBS and blocked with goat serum, the slides were incubated in primary antibodies at 4°C for over 12 h. Rinsed with PBS, the slides were incubated in Alexa Fluor-conjugated CY3- or 488-secondary antibodies (1 : 5000, G-21234, Thermo Fisher) for 1 h at room temperature. Finally, after being rinsed with PBS, the slides were counterstained with the nuclear marker DAPI and wet mounted. All images were obtained through a fluorescence microscope (Nikon TE2000-U, Japan), and the fluorescence intensity was quantified using ImageJ.

### 2.6. Tubular Injury and Cell Apoptosis

PAS staining was used to detect the cellular injury of renal tubules. The percentage of injured tubules was determined in 10 random fields at ×400 magnification in each section. Additionally, TUNEL assay was conducted to examine apoptotic cells in the renal tissue using the In Situ Cell Death Detection Kit (Roche, Switzerland). Positive cells in the TUNEL assay were determined in 10 random fields at ×400 magnification for every section.

### 2.7. qRT-PCR

Total RNA was acquired from THP-1 and HK-2 cells by TRIzol (Invitrogen, USA). Then, cDNAs were synthesized from 2 *μ*g of total RNA by the PrimeScript RT Reagent Kit (TaKaRa). qPCR was conducted by SYBR green qPCR master mix (QIAGEN, Germany) via an ABI Prism 7300 system. All the reactions were triplicated. Target gene expression levels were quantified using the double-delta method (2^–ΔΔ*Ct*^) for 3 independent experiments with normalization. Primers (provided by TSINGKE) used in experiments are listed in Table 1.

### 2.8. Western Blot

Whole-protein extracts were obtained with sonication and separated on 10% SDS-PAGE and then were transferred onto PVDF membrane (Millipore, USA). Blocked with TBST (pH 7.5) containing 5% BSA, the membranes were incubated within primary antibodies against MCP1 (1 : 1200, DF7577, Affinity), iNOS (1 : 800, AF0199, Affinity), Arg1 (1 : 800, DF6657, Affinity), IL-1*β* (1 : 800, AF5103, Affinity), phosphorylated NF-*κ*Bp65 (1 : 1000, 3039, CST), NF-*κ*Bp65 (1 : 1000, 8242, CST), PPAR*γ* (1 : 600, 2435, CST), HO-1 (1 : 1000, AF5393, Affinity), Nrf2 (1 : 1000, AF0639, Affinity), SOD1 (1 : 1000, AF5198, Affinity), the endogenous control *β*-actin (1 : 1000, 4970, CST), and GAPDH (1 : 1000, 3683, CST) at 4°C overnight. The membranes were then incubated in HRP-conjugated secondary antibodies (1 : 3000, 7074, CST) for 1 h at room temperature. We used the ECL Western Blot Kit (Thermo Scientific Pierce) to examine the bands and scanned them using a LAS4000 analyzer (GE Healthcare). The immunoblot density was examined by ImageJ and normalized by *β*-actin or GAPDH.

### 2.9. Enzyme-Linked Immunosorbent Assay (ELISA)

Cell supernatants were obtained after centrifugation and were stored at −80°C until further use. Secretion levels of TNF-*α*, IL-4, IL-6, and IL-10 were detected via specific ELISA kits, according to the instructions from the manufacturer (Dakewe, Shenzhen, China).

### 2.10. Measurement of Renal and Intracellular ROS Levels

ROS levels were assessed by the ROS Assay Kit containing 2,7-dichlorodi-hydrofluorescein-diacetate (DCFH-DA) (Nanjing Jiancheng Bioengineering Institute, China). Fluorescence intensities of DCFH-DA were measured by the fluorescence microplate for kidney tissues and by a fluorescence microscope for cells.

### 2.11. Measurement of Hydrogen Peroxide (H_2_O_2_), Malondialdehyde (MDA), and Glutathione (GSH)

H_2_O_2_, MDA, and GSH activities in the renal tissues or cell supernatants were measured by the Hydrogen Peroxide Assay Kit, Lipid Peroxidation MDA Assay Kit, and GSH Assay Kit (Beyotime, China), respectively, according to instructions from manufacturers. All results were normalized according to corresponding control samples.

### 2.12. Lactate Dehydrogenase (LDH) Assay

Cellular LDH activities were detected using the LDH Assay Kit (Beyotime, China), according to the direction from the manufacturer. The absorbance (490 nm) was measured by a microplate reader (Thermo Multiskan MK3, USA).

### 2.13. Flow Cytometry

Cell death was measured through the Annexin V-FITC/PI (fluorescein isothiocyanate/propidium iodide) Apoptosis Detection Kit (R&D Systems, USA), according to the direction from the manufacturer. The fluorescence intensity of Annexin V-FITC/PI was detected through flow cytometry (BD Bioscience).

For quantifying the renal macrophage polarization in mouse kidneys, kidney tissues were minced into 1 mm^3^ fragments and then digested in RPMI 1640 buffer containing 100 U/mL DNase I and 2 mg/mL collagenase type D for 60 min at 37°C and then passed through a 70 *μ*m mesh to get single-cell suspension. Red blood cell lysis buffer (Sigma, USA) was used for lysing the red blood cells in the suspension. Mps were centrifuged and then resuspended in FACS buffer on ice. Incubated with 2.5 *μ*g/mL Fc-blocking solution, Mps were resuspended in FACS buffer. Then, 10^6^ cells were stained with 3 fluorochrome-labeled antibodies: F4/80 (eBioscience)-PE, CD11c-FITC, and CD206-FITC. Finally, Mps were detected immediately on a FACS Canto II cytometer with DIVA software (Becton Dickinson). The data were analyzed by Cyflogic V.1.2.1 software.

### 2.14. Crystal Adhesion Assay

HK-2 cells were cultured to 100% confluency in a 6-well plate. Stimulated with COM crystal and ROSI or/and GW9662 treatment for 48 h, the plate was thoroughly washed 3 times using PBS to remove unbound crystals from cells. The crystal quantity was examined using a microscope. Images were randomly selected from 10 visual fields (magnification of ×400) and quantified using ImageJ Pro Plus software [29]. All experimental group data were normalized to the normal control group based on 3 independently repeated experiments.

### 2.15. Small Interfering RNA Knockdown

For cell transfections, Nrf2 siRNA was obtained from Santa Cruz Biotechnology (Ribo, Guangzhou, China). HK-2 cells were transfected with 50 nM siRNA with Lipofectamine 2000 siRNA transfection reagent (Thermo Fisher) for 24 h before ROSI treatment.

### 2.16. Statistical Analysis

Data were presented as mean ± standard deviation (SD). A two-tailed *t*-test was conducted to identify statistical difference using GraphPad Prism 6.0 (GraphPad Software, USA). Difference was considered statistically significant at *p* < 0.05.

## 3. Results

### 3.1. ROSI Suppressed the Deposition of Renal CaOx Crystals in the Mouse Model

**Figure 1:**
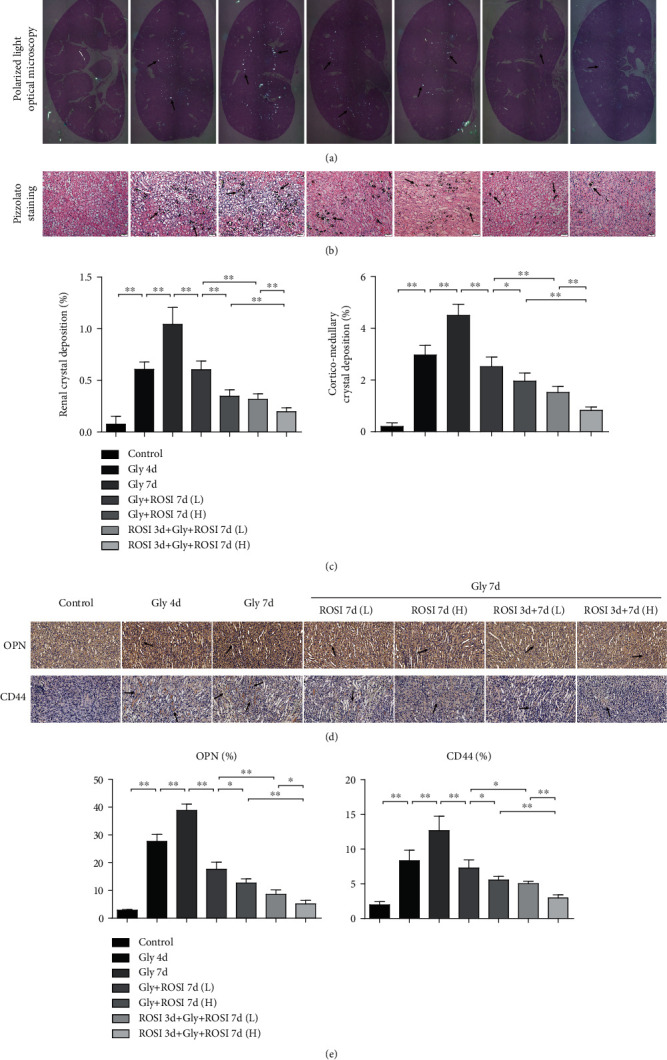
ROSI decreased the deposition of renal CaOx crystal in the mouse model. (a) Polarized light optical microscopy (arrows). ×20 magnification. (b) Pizzolato staining. Pizzolato staining indicates CaOx crystals (arrows). Scale bar = 50 *μ*m. (c) The proportion of the crystal deposition area in the kidney and the proportion of crystal deposition areas in the corticomedullary border. (d) Immunohistochemical distribution of crystal-related gene OPN and crystal adhesion-related gene CD44. Scale bar = 50 *μ*m. (e) The proportion of the IHC-positive area. Gly: glyoxylic acid; ROSI: rosiglitazone; CaOx: calcium oxalate. ^∗^*p* < 0.05; ^∗∗^*p* < 0.01.

Renal CaOx crystals were measured by polarized light microscopy and Pizzolato straining. The results revealed that crystals were mainly deposited in renal tubules at the corticomedullary borders and the number of crystals gradually increased over time in the Gly groups (Figures 1(a) and 1(b)). Crystals in the entire kidney or renal corticomedullary borders were significantly larger and more in the Gly 7d group than in the Gly 4d group. Notably, crystals were significantly decreased in ROSI treatment groups than in the Gly 7d group in a dose-dependent way. Furthermore, there were significantly fewer and smaller crystals in the ROSI pretreatment groups than in the nonpretreatment groups (Figure 1(c)).

IHC results suggested that the expression levels of the crystal-related gene osteopontin (OPN) and crystal adhesion-related gene CD44 were markedly increased in the Gly 4d group than in the control group, increased in the Gly 7d group than in the Gly 4d group, dose dependently decreased in the ROSI treatment groups than in the Gly 7d group, and decreased in the pretreatment groups than in the nonpretreatment groups (Figures 1(d) and 1(e)).

### 3.2. ROSI Decreased Renal Cell Apoptosis, Tubular Injury, and Proinflammatory Response in the Mouse Model

**Figure 2:**
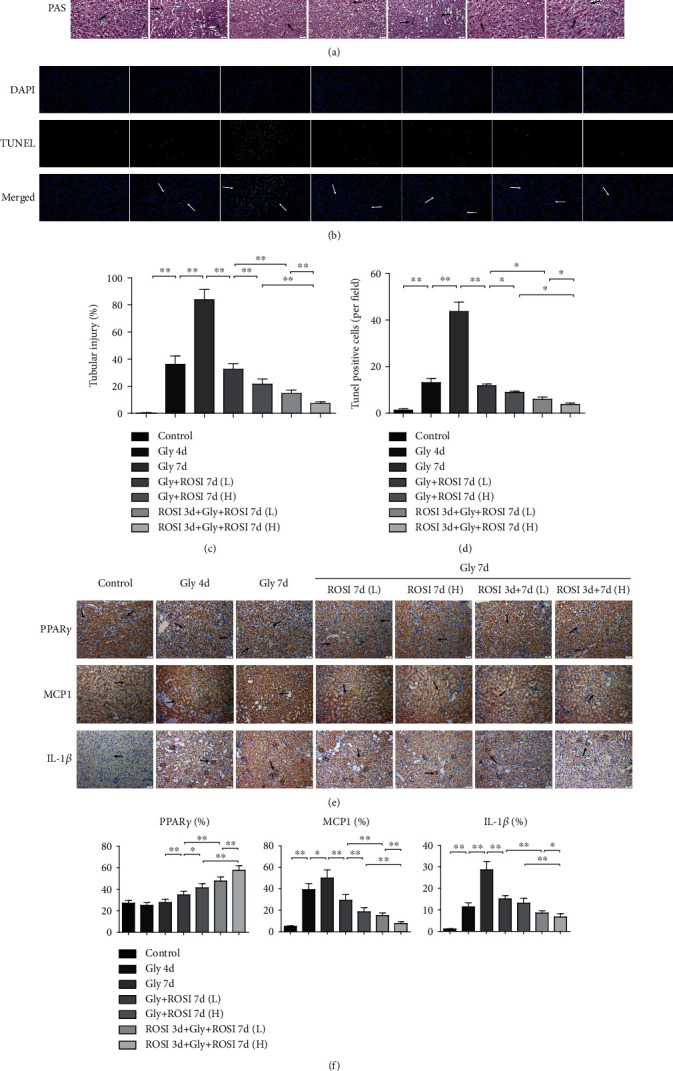
ROSI decreased renal tubular injury, cell apoptosis, and proinflammatory response in the mouse model. (a) PAS staining. PAS staining denotes tubular injury (arrows). Scale bar = 50 *μ*m. (b) Cell apoptosis in the kidneys (arrows). Scale bar = 50 *μ*m. (c) The percentage of damaged tubules displayed in PAS staining. (d) The mean number of apoptotic cells per high-power field (×400; *n* = 10 fields per section) in the TUNEL assay. (e) Immunohistochemical distribution of genetic expression of PPAR*γ*, Mps-related molecule MCP1, and proinflammatory cytokine IL-1*β*. Scale bar = 50 *μ*m. (f) The proportion of the IHC-positive area. Gly: glyoxylic acid; ROSI: rosiglitazone; PAS: periodic acid–Schiff; IL-1*β*: interleukin-1*β*; MCP1: monocyte chemotactic protein-1; PPAR*γ*: peroxisome proliferator-activated receptor *γ*; IHC: immunohistochemistry. ^∗^*p* < 0.05; ^∗∗^*p* < 0.01.

For all groups, PAS staining showed that positively stained cells were mainly located in the renal tubules. Positive tubules were significantly fewer in the Gly 7d group and markedly more in the ROSI-treated groups than in the Gly 7d group in a dose-dependent way. In addition, positively stained tubules were substantially more in the pretreatment groups than in the nonpretreatment groups (Figures 2(a) and 2(c)).

TUNEL assay results revealed that positive cells were significantly more in the Gly 7d group, but fewer in the ROSI treatment groups than in the Gly 7d group, presenting a dose-dependent reduction. Moreover, positive cells were significantly fewer in the ROSI pretreatment groups than in the nonpretreatment groups (Figures 2(b) and 2(d)).

The expression of renal PPAR*γ* was markedly and dose dependently increased in the ROSI treatment groups than in the Gly 7d group, and PPAR*γ* expression was markedly increased in the ROSI pretreatment groups than in the nonpretreatment groups. The expression levels of the Mps-related molecule MCP1 and proinflammatory cytokine IL-1*β* were markedly increased in the Gly 4d and 7d groups; however, they were markedly decreased in the ROSI treatment groups in a dose-dependent manner. A significant decrease in expression levels of MCP1 and IL-1*β* was also noted in the pretreatment groups (vs. nonpretreatment groups) (Figures 2(e) and 2(f)).

### 3.3. ROSI Decreased Renal Oxidative Stress in the Mouse Model

**Figure 3:**
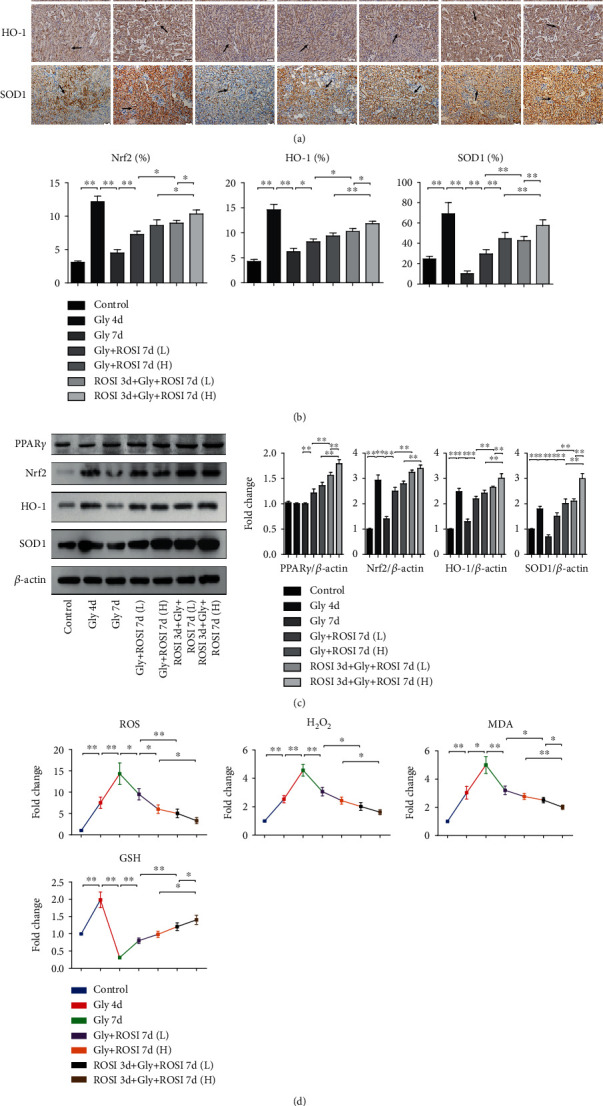
ROSI inhibited renal oxidative stress in the mouse model. (a) Immunohistochemical distribution of gene expression of Nrf2, HO-1, and SOD1 in the kidneys (arrows). Scale bar = 50 *μ*m. (b) The proportion of the IHC-positive area. (c) The expression of PPAR*γ*, Nrf2, HO-1, and SOD1 in kidney tissues by Western blotting. (d) ROS, H_2_O_2_, MDA, and GSH levels in mouse renal tissues. Gly: glyoxylic acid; ROSI: rosiglitazone. ^∗^*p* < 0.05; ^∗∗^*p* < 0.01.

Our results demonstrated that the expression of Nrf2, HO-1, SOD1, and GSH markedly increased in the Gly 4d group than in the control group, decreased in the Gly 7d group than in the Gly 4d group, dose dependently increased in the ROSI treatment groups than in the Gly 7d group, and increased in the pretreatment groups than in the nonpretreatment groups. The expression of PPAR*γ* was markedly and dose dependently increased in the ROSI treatment groups than in the Gly 7d group and was markedly increased in the ROSI pretreatment groups than in the nonpretreatment groups (Figure 3).

The generations of ROS, H_2_O_2_, and MDA markedly increased in the Gly 4d group than in the control group, increased in the Gly 7d group than in the Gly 4d group, dose dependently decreased in the ROSI treatment groups than in the Gly 7d group, and decreased in the pretreatment groups than in the nonpretreatment groups (Figure 3(c)).

### 3.4. ROSI Suppressed Oxidative Stress Injury via Promoting the Nrf2/HO-1 Pathway in HK-2 Cell

**Figure 4:**
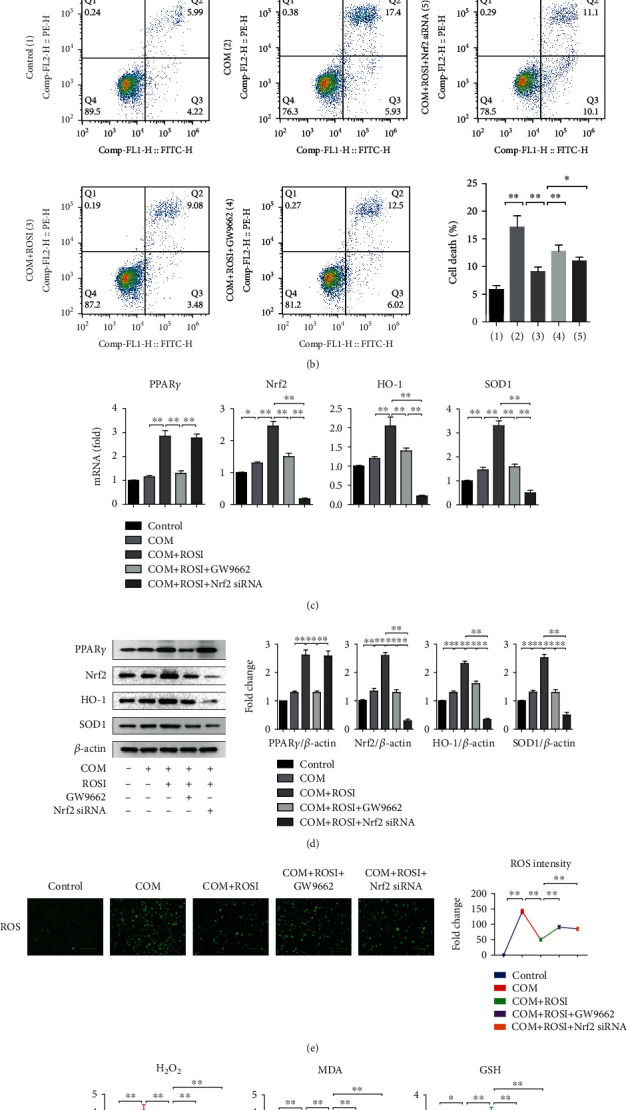
ROSI suppressed CaOx-induced oxidative stress injury and promoted the Nrf2/HO-1 pathway in HK-2 cell. (a) Cellular LDH levels in HK-2 cells. (b) HK-2 cell death by flow cytometry. (c) mRNA expression levels of Nrf2, HO-1, and SOD1 in HK-2 cells by qRT-PCR. (d) The expression of PPAR*γ*, Nrf2, HO-1, and SOD1 in HK-2 cells by Western blot. (e) Detection of intracellular ROS levels by a fluorescence microscope. (f) H_2_O_2_, MDA, and GSH levels in HK-2 cells. COM: calcium oxalate monohydrate; ROSI: rosiglitazone. ^∗^*p* < 0.05; ^∗∗^*p* < 0.01.

For exploring the effect of ROSI on tubular epitheliums, we carried out cell experiments. The results revealed that LDH activity and death of HK-2 cells markedly increased in the COM group compared to the control group. Moreover, LDH activity and cell death markedly decreased after simultaneous ROSI treatment than in the COM group and increased in the GW9662 treatment group than in the ROSI group. In addition, Nrf2 silencing by siRNA also attenuated the effect of ROSI treatment (Figures 4(a) and 4(b)).

In HK-2 cells, expression of PPAR*γ* markedly increased in the ROSI treatment group and markedly decreased in the GW9662-treated group, whereas it was not significantly affected by Nrf2-siRNA treatment. Genetic expression of Nrf2, HO-1, SOD1, and GSH markedly increased in the COM group than in the control group, increased in the ROSI group than in the COM group, and decreased in the GW9662 and Nrf2-siRNA treatment groups than in the ROSI group (Figures 4(c), 4(d), and 4(f)).

Furthermore, the productions of ROS, H_2_O_2_, and MDA in HK-2 cells markedly increased in the COM group than in the control group, decreased in the ROSI treatment group than in the COM group, and increased in the GW9662 and Nrf2-siRNA treatment groups than in the ROSI group (Figures 4(e) and 4(f)).

### 3.5. ROSI Decreased Macrophage Recruitment and Polarization of M1Mps but Increased Polarization of M2Mps in the Mouse Model

**Figure 5:**
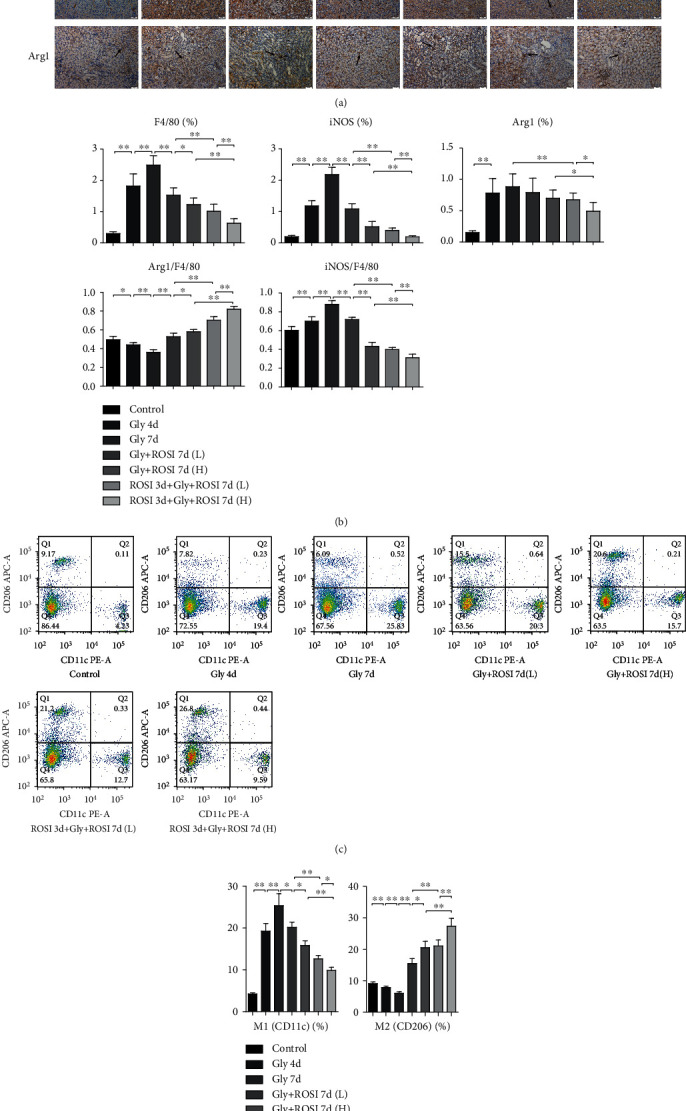
ROSI decreased renal macrophage recruitment and M1Mp polarization and promoted renal M2Mp polarization in the mouse model. (a) Immunohistochemical distribution of genetic expression of PPAR*γ*, macrophage-related molecules, and proinflammatory cytokines (arrows). Scale bar = 50 *μ*m. (b) The proportion of the IHC-positive area and the ratio of Arg1/F4/80 and iNOS/F4/80. (c) Flow cytometric detection of Mps in kidney tissues. (d) The proportion of M1Mps and M2Mps in kidney tissues. Gly: glyoxylic acid; ROSI: rosiglitazone; PPAR*γ*: peroxisome proliferator-activated receptor *γ*. ^∗^*p* < 0.05; ^∗∗^*p* < 0.01.

IHC showed that expression levels of Mp marker F4/80, M1Mp marker iNOS, and M2Mp marker Arg1 were markedly increased in the Gly 4d and 7d groups. However, these levels were markedly decreased in the ROSI treatment groups in a dose-dependent way, with a significant reduction observed in the ROSI pretreatment groups than in the nonpretreatment groups (Figures 5(a) and 5(b)).

Interestingly, the ratio of Arg1/F4/80 markedly decreased in the Gly 4d and 7d groups than in the control group, with a dose-dependent increase observed in the ROSI-treated groups. In addition, the ratio of Arg/F4/80 was higher in the ROSI pretreatment groups than in the nonpretreatment groups. Meanwhile, the ratio of iNOS/F4/80 demonstrated the opposite trend (Figure 5(b)).

Furthermore, the flow cytometry results of renal macrophage polarization showed that the proportion of M1Mps markedly increased in the Gly 4d and 7d groups than in the control group, with a dose-dependent decrease in the ROSI treatment groups. Meanwhile, the proportion of M1Mps markedly decreased in the ROSI pretreatment groups than in the nonpretreatment groups, whereas the proportion of M2Mps demonstrated the opposite trend (Figures 5(c) and 5(d)).

### 3.6. ROSI Declined COM-Stimulated M1Mp Polarization and Crystal Adhesion, as well as Promoted M2Mp Polarization In Vitro

**Figure 6:**
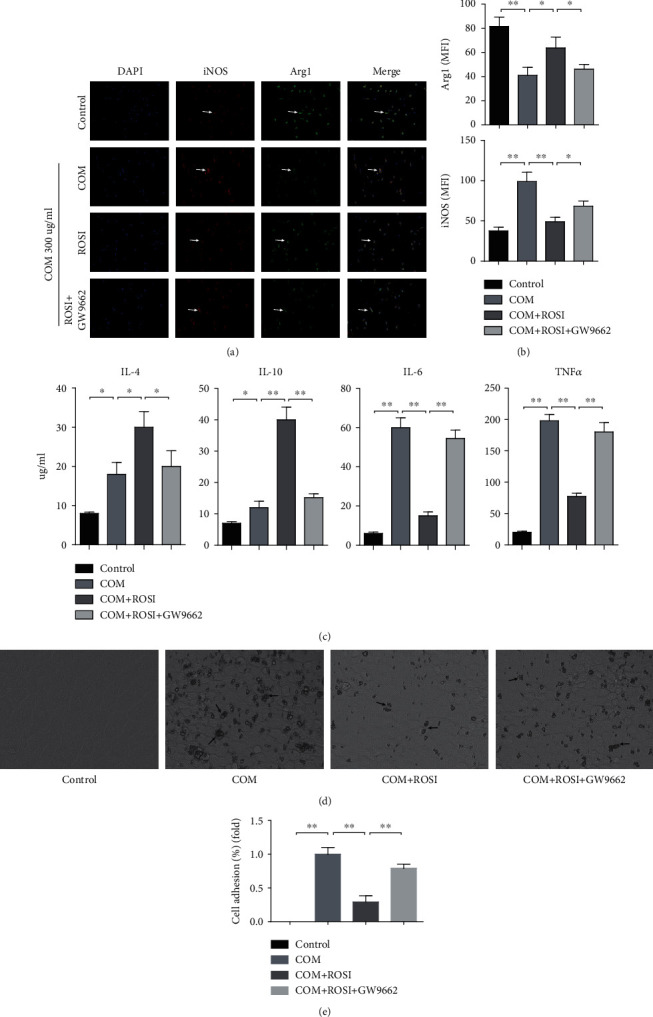
ROSI declined COM-stimulated M1Mp polarization and crystal adhesion and promoted M2Mp polarization in vitro. (a) Fluorescence immunohistochemical distribution of Arg1 (green) and iNOS (red) in THP-1 cells with the stimulus of COM or ROSI (1 *μ*M) or GW9662 (10 *μ*M) (arrows). (b) Evaluation of mean fluorescence intensity (MFI) of Arg1 and iNOS. (c) ELISA. The expression levels of IL-4, IL-10, IL-6, and TNF-*α*. (d) Crystal adhesion assay. COM crystal adhesion to HK-2 cells was observed (arrows). COM: calcium oxalate monohydrate; ROSI: rosiglitazone; iNOS: induced nitric oxide synthase; TNF-*α*: tumor necrosis factor *α*. ^∗^*p* < 0.05; ^∗∗^*p* < 0.01.

The experiments revealed that ROSI treatment upregulated renal PPAR*γ* expression, inhibited Mp aggregation, and shifted the COM-stimulated M1Mp polarization to M2Mp polarization. Moreover, compared with the control group, THP-1 cells treated with COM primarily showed M1Mp polarization, characterized by increased expression of M1Mp marker iNOS and proinflammatory cytokines (TNF-*α* and IL-6), but decreased expression of M2Mp marker Arg1 and anti-inflammatory cytokines (IL-4 and IL-10) (Figures 6(a) and 6(b)).

**Figure 7:**
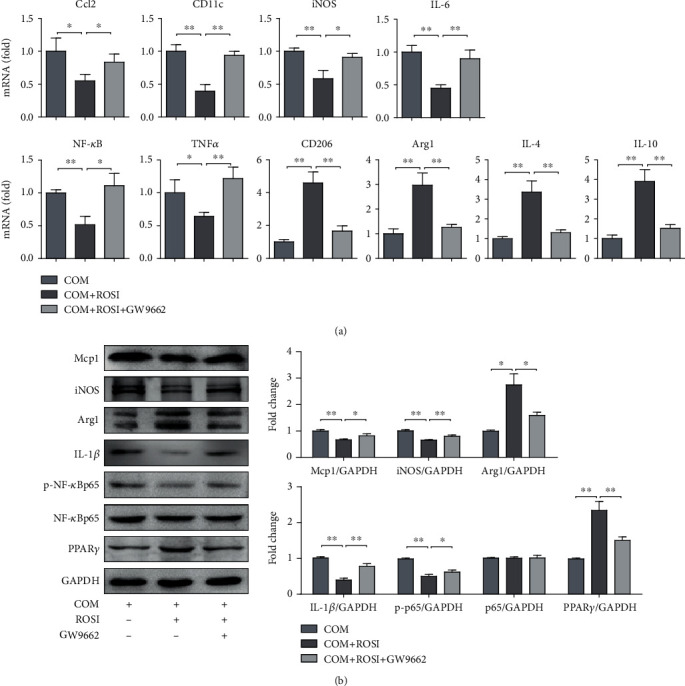
Genetic expression of macrophage-related molecules, proinflammatory cytokines, and anti-inflammatory cytokines. (a) mRNA levels in THP-1 cells stimulated with COM or ROSI (1 *μ*M) or GW9662 (10 *μ*M) by qRT-PCR. (b) Genetic expression determined by Western blot. COM: calcium oxalate monohydrate; ROSI: rosiglitazone. ^∗^*p* < 0.05; ^∗∗^*p* < 0.01.

Following COM stimulation, THP-1 cells were treated with ROSI (PPAR*γ* agonist, 1 *μ*M) and with or without GW9662 (PPAR*γ* antagonist, 10 *μ*M). The results showed that ROSI significantly decreased the expression of PPAR*γ*, M1Mps markers (iNOS and CD11c), proinflammatory cytokines (Ccl2, IL-6, and TNF-*α*), phosphorylated NF-*κ*B and IL-1*β*, but increased the expression of M2Mp markers (Arg1 and CD206) and anti-inflammatory cytokines (IL-4 and IL-10). However, GW9662 diminished the effects of ROSI (Figures 6 and 7). Moreover, as for the COM crystal adhesion to HK-2 cells, ROSI significantly decreased crystal adhesion, whereas GW9662 diminished the effect of ROSI (Figures 6(d) and 6(e)).

## 4. Discussion

Crystal deposition may result in tubular injury [30], which in turn promotes crystal deposition [31]. In the above process, oxidative stress and inflammatory response play pivotal roles [32].

Being exposed to CaOx crystals, tubular epitheliums overproduce oxidative stress products, such as ROS [6]. As a leading mediator of oxidative stress, ROS leads to tubular injury, promoting the adhesion and deposition of crystals [9]. Nrf2 is a redox-sensitive transcription factor, playing critical roles in reducing intracellular oxidative stress and tissue injury [33]. Previous reports have revealed that the Nrf2/HO-1 pathway has potent antioxidant effects [21], whereby inhibiting the formation of CaOx-induced nephrolithiasis [34, 35].

Deposition of THE CaOx crystal can pose migration and aggregation of Mps to the areas of crystal deposition [36]. Polarization of Mps is deeply involved in inflammation regulation, crystal phagocytosis, and crystal removal [37]. A previous study has revealed that CaOx crystals effectively induce M1Mp polarization, promoting renal injury and crystal deposition [38]. Furthermore, Taguchi et al. have observed that M2Mps have the ability of crystal phagocytosis and antiadherence [17]. As an agonist of PPAR*γ*, ROSI takes a vital part in the intricate modulatory network of alternative activation of Mps [26]. Tabassum and Mahboob found that ROSI could reduce HFD-induced oxidative stress damage [39]. Deng et al. revealed that ROSI treatment inhibited renal macrophage infiltration and TGF-*β* and NF-*κ*B pathway activation [40]. Liu et al. found that ROSI could regulate the TGF-*β*1 and HGF/c-Met pathways to play an antioxidant effect to reduce crystal deposition in the hyperoxaluria rat model [41]. In addition, previous studies have suggested that pioglitazone, another PPAR*γ* agonist, can suppress crystal deposition, thus exhibiting prominent anti-inflammatory ability [23, 42, 43].

In this study, we observed that ROSI could suppress oxidative stress injury by upregulating the Nrf2/HO-1 pathway. Moreover, we noted that ROSI could regulate inflammation by shifting COM-stimulated M1Mp polarization toward M2Mp polarization. Namely, the above effects might suppress tubular injury synergistically, consequently reducing the deposition of crystal.

As the above speculated, our results indicated that the deposited crystals were fewer and smaller and the renal injury was also milder in the ROSI-treated groups; these effects were more pronounced in the groups with ROSI pretreatment. Furthermore, the apoptosis of HK-2 cells was significantly reduced after treating with ROSI.

Moreover, our finding revealed that the generation of oxidative stress products, i.e., ROS, MDA, and H_2_O_2_, was significantly reduced. Conversely, expression levels of antioxidant products, i.e., SOD1 and GSH, were significantly upregulated after treating with ROSI. In addition, the effects of ROSI for suppressing oxidative stress and inflammation were found to be dose dependent both *in vivo* and *in vitro*.

Finally, in ROSI-treated hyperoxaluria mice, the expression of renal PPAR*γ* was markedly increased. Subsequently, the MCP1 expression and Mp recruitment reduced with the decrease in M1Mps and the increase in M2Mps. The expression of iNOS was increased, but the expression of Arg1 was decreased in the Gly 7d group, which is similar to findings of a microarray analysis of renal papillary tissues from nephrolithiasis formers [13]. Interestingly, the renal Mps mainly were polarized into M1Mps after being stimulated by CaOx crystals. In contrast, the proportion of M2Mps increased in the ROSI-treated groups, reaching up to 70%. Accordingly, ROSI may pose a shift of Mp polarization. In vitro, ROSI also exhibited an anti-inflammatory ability due to shifting polarization of Mps.

We speculate that ROSI upregulates the expression of PPAR*γ*, whereby it exerts antioxidant and anti-inflammatory functions, which subsequently synergistically suppressed CaOx crystal deposition and renal injury. In terms of oxidative stress, ROSI potentially improves the imbalance between oxidative and antioxidant products by upregulating the Nrf2/HO-1 pathway. Finally, in terms of inflammation, M1Mp polarization was reduced while M2Mp polarization was enhanced and, in turn, lowered the expression of MCP1 in the renal tubular epitheliums and proinflammatory cytokines from Mps; this contributed to a subsequent decrease in macrophage recruitment and renal inflammation.

It should be noted that several limitations exist in this research: (1) with pronounced high concentration of oxalate, the mouse model used in in vivo experiment fails to mimic the development of CaOx nephrolithiasis comprehensively; (2) the doses of ROSI (2.5 mg/kg, 5 mg/kg) used in mouse model were much higher than those employed in clinical settings (usually, 4 mg/d); (3) the effects of ROSI are indirect and gentle, which may be influenced by other latent factors; and (4) the preventive and therapeutic effects, as well as the safety of ROSI for clinical practice, need further confirmation by well-designed clinical researches.

## 5. Conclusion

**Figure 8:**
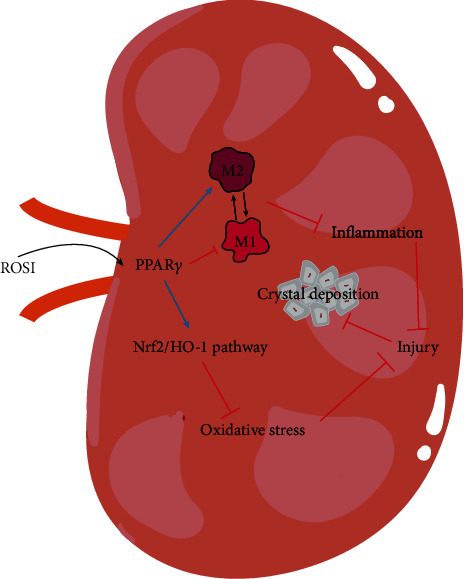
Mechanisms by which ROSI suppresses CaOx crystal deposition. ROSI: rosiglitazone; PPAR*γ*: peroxisome proliferator-activated receptor *γ*.

Our results reveal that ROSI might decrease tubular injury-induced renal CaOx crystal deposition via suppressing oxidative stress and inflammation, mediated by promoting the Nrf2/HO-1 pathway and shifting macrophage polarization (Figure 8). Thus, ROSI could be a potential preventive and therapeutic drug for CaOx nephrolithiasis.

## Figures and Tables

**Table 1:** Primers.

Primer (human)	Forward (5′-3′)	Reverse (5′-3′)
Ccl2	AGAATCACCAGCAGCAAGTGTCC	TCCTGAACCCACTTCTGCTTGG
CD11c	GATGCTCAGAGATACTTCACGGC	CCACACCATCACTTCTGCGTTC
iNOS	GCTCTACACCTCCAATGTGACC	CTGCCGAGATTTGAGCCTCATG
IL-6	GCACTGGCAGAAAACAACCT	TCAAACTCCAAAAGACCAGTGA
NF-*κ*B	TGAACCGAAACTCTGGCAGCTG	CATCAGCTTGCGAAAAGGAGCC
TNF-*α*	CCCAGGGACCTCTCTCTAATC	ATGGGCTACAGGCTTGTCACT
CD206	CCGTATGCCGGTCACTGTTA	CAATTCCTCGATGGTGTGGA
Arg1	GTGGAAACTTGCATGGACAAC	AATCCTGGCACATCGGGAATC
IL-4	ATGGGTCTCACCTCCCAACT	GATGTCTGTTACGGTCAACTCG
IL-10	GACTTTAAGGGTTACCTGGGTTG	TCACATGCGCCTTGATGTCTG
IL-1*β*	ACGCTCCGGGACTCACAGCA	TGAGGCCCAAGGCCACAGGT
Nrf2	CACATCCAGTCAGAAACCAGTGG	GGAATGTCTGCGCCAAAAGCTG
HO-1	CCAGGCAGAGAATGCTGAGTTC	AAGACTGGGCTCTCCTTGTTGC
SOD1	CTCACTCTCAGGAGACCATTGC	CCACAAGCCAAACGACTTCCAG
PPAR*γ*	CATGGCAATTGAATGTCGTGTC	CCGGAAGAAACCCTTGCAT
GAPDH	GACCTGACCTGCCGTCTA	AGGAGTGGGTGTCGCTGT

## Data Availability

The data on supporting information are available from the corresponding author upon reasonable request.

## Abbreviations

Mps: Macrophages
ROSI: Rosiglitazone
PPAR*γ*: Peroxisome proliferator-activated receptor *γ*
CaOx: Calcium oxalate
ROS: Reactive oxygen species
Gly: Glyoxylic acid
COM: Calcium oxalate monohydrate
MCP1: Monocyte chemotactic protein 1
H_2_O_2_: Hydrogen peroxide
MDA: Malondialdehyde
GSH: Glutathione
LDH: Lactate dehydrogenase.

## Additional Points

*Highlights*. Rosiglitazone suppresses renal crystal deposition in the hyperoxaluria mouse model. Rosiglitazone ameliorates renal epithelial oxidative and inflammatory injury in the hyperoxaluria mouse model. Rosiglitazone is a potential preventive and therapeutic drug for CaOx nephrolithiasis.
